# Single-level laminoplasty approach to selective dorsal rhizotomy with conus localization by rapid spine MRI

**DOI:** 10.1007/s00381-024-06439-z

**Published:** 2024-05-27

**Authors:** John P. Andrews, Cecilia Dalle Ore, Joseph Falcone, Melessa Hirschhorn, Courtney Sagar, Kathryn Sigford, Yumi Mitsuya, Taylor Chung, Peter P. Sun

**Affiliations:** 1 Department of Neurological Surgery, University of California-San Francisco, San Francisco, CA 94143 USA; 2 Department of Neurological Surgery, UCSF Benioff Children’s Hospital Oakland, Oakland, USA; 3 Department of Physical Medicine and Rehabilitation, UCSF Benioff Children’s Hospital Oakland, Oakland, USA; 4 Department of Diagnostic Imaging, UCSF Benioff Children’s Hospital Oakland, Oakland, USA

**Keywords:** Selective dorsal rhizotomy, Rapid MRI, Spinal MRI, Single-shot sequence

## Abstract

**Introduction:**

While selective dorsal rhizotomy (SDR) was originally described as a multilevel approach, single-level approaches are now popularized. Conus localization is beneficial for operative planning in single-level selective dorsal rhizotomy. Our approach to SDR involves minimal exposure for a single-level laminoplasty, preserving one attached interspinous ligament. Pre-operative conus localization is required for this tailored approach to determine the laminoplasty level and dictate rostral or caudal division of the superior spinus ligament. While rapid MRI sequences have been popularized for pediatric cranial imaging, its utility for spinal imagining is less well-described, and specific application for conus localization has not been reported.

**Objective:**

Illustrate that rapid MRI without sedation is sufficient to identify conus level for tailored single-level laminoplasty SDR.

**Material and methods:**

Patients undergoing SDR from 2014 to 2022 at one institution were reviewed for type of pre-operative MRI (rapid vs full), conus level, procedural time for MRI, and radiology report. The typical rapid MRI has four sequences utilizing single-shot technique (scout, sagittal T2, axial T2, and axial T1) that typically take less than 1 min each of acquisition time, with non-single-shot sequences added periodically in cooperative patients. To include time for patient positioning, pre-scan shimming, procedural incidentals, and other patient-specific variations, MRI procedure length was recorded as documented in the electronic medical record.

**Results:**

*N* = 100 patients had documentation of an MRI for pre-operative imaging. Seventy-nine of these had a rapid MRI, and 21 required a full MRI with anesthesia for their treatment plan. Mean total procedure time for rapid MRI was 21.5 min (median 17). Mean procedure time for MRI under general anesthesia was 91.2 min (median 94). Of patients with rapid MRI imaging, 2/79 had an ambiguous conus level (1 from motion artifact, 1 from spinal hardware) vs 1/21 with a full MRI under anesthesia (due to spinal hardware).

**Conclusion:**

Rapid spinal MRI without sedation can be used for conus localization in a pediatric population. This may be routinely used as pre-operative imaging for a single-level approach to selective dorsal rhizotomy, without sedation or intubation procedures.

## Introduction

Selective dorsal rhizotomy (SDR) is an effective treatment for spasticity and improves gross motor function in select patients [1, 2]. Surgical techniques used for this procedure have evolved from multilevel-laminectomies [3] to minimal exposure techniques such as single-level laminectomy and laminoplasty at the conus, as opposed to the nerve roots [4–6].

Surgical techniques aiming to minimize exposure allow for smaller incisions, less boney disruption, and less muscle dissection. A laminoplasty approach aims to preserve the integrity of dorsal elements where possible. Such limited exposure approaches benefit from pre-operative imaging to delineate conus level, which may vary in the population [7]. While intra-operative ultrasound may be used prior to incision in young children, this can be complicated and less effective in patients with larger habitus. By contrast, conventional MRI imaging reliably determines level of the conus. Conventional MRI studies, however, are long and require cooperation from patients to avoid movement artifact. Many pediatric patients qualifying for SDR have difficulty cooperating with standard MRI studies without sedation or general anesthesia [8].

Parents and caregivers often raise concerns about anesthetic exposure in pediatric patients, so the option to avoid sedation the in pre-operative work-up can be a valuable way to address these concerns. Conventional spinal MRI also requires significant investment of time by the patient and their caregivers and coordination with anesthesia providers’ schedules. An MRI technique that identifies the conus level, while avoiding the time and additional sedation required for standard spinal MRI studies would be optimal.

Rapid MRI for cranial imaging is widely used for pediatric neurosurgical indications. These rapid acquisition sequences avoid the radiation of computed tomography scans while maintaining the ability for expedient, detailed imaging [9–11]. Compared to cranial rapid MRI sequences in pediatric patients [12–14], literature on rapid MRI for spinal imaging is sparse [15]. We sought to evaluate these rapid sequence MRI techniques in the specific context of conus localization for pre-operative work-up in a cohort of patients undergoing single-level laminoplasty for SDR.

The surgical technique of choice at the authors’ institution is a single-level laminoplasty at the level of the conus, and standard pre-operative work-up includes a rapid MRI of the spine to localize conus for planning the laminoplasty level. Our laminoplasty technique involves minimizing interspinous ligament disruption by dividing at only one location (either cranial or caudal to the laminoplasty level) and reflecting the laminoplasty away from this division during the surgery. The exposure requires conus localization.

Here, we present a series of patients who underwent single-level laminoplasty for SDR, comparing those who had a rapid MRI without sedation to those who had a full MRI of the spine with sedation for pre-operative conus localization.

## Methods

### Chart review

We reviewed the charts of patients undergoing single-level laminoplasty for selective dorsal rhizotomy at one institution between the years 2014–2022. Data recorded included pre-operative MRI type (rapid vs full), conus level on radiology report, age, pre-operative Gross Motor Function Classification System (GMFCS) score and procedural time for MRI.

#### Rapid MRI

Imaging was performed on a Philips Intera 1.5 T MR scanner on R3.2 software (Philips Healthcare, Best, The Netherlands). The MR technique for the rapid spine images is routinely available standard pulse sequence on all MR scanners by all MR vendors. For the typical rapid MRI, after initial survey scan, the three pulse sequences were utilized: single-shot turbo spin echo (fast spin echo) T2-weighted sagittal and axial and single-shot T1-weighed fast gradient echo axial. The T2-weighted sagittal images were acquired with in-plane resolution of 0.9 × 1.2 mm, 4-mm thick slice, overlapping slices with skip of − 2 mm (reconstructed to 0.6 × 0.6 × 4 mm voxels); the T2-weighed axial images were acquired with in-plane resolution of 0.9 × 1.2 mm, 6-mm-thick slice, contiguous slices with no skip (reconstructed to 0.6 × 0.6 × 6 mm voxels); the T1-weighted axial images were acquired with in-plane resolution of 1.2 × 1.7 mm, 4 mm thick, contiguous slices with no skip (reconstructed to 1.2 × 1.2 × 4 mm voxels). No parallel imaging acceleration was applied to cut down on pre-scan preparation time. These imaging techniques are referred to in short-hand as single-shot (SSH) sequences.

While SSH sequences take less than 1 min each [8, 15], with sub-second acquisition speed per image to freeze motion, MRI procedure length was recorded as documented in the electronic medical record to include time for patient positioning, procedural incidentals, and other patient-specific variations. During rapid MRI procedures where patients were cooperative, one or two non-single-shot (non-SSH) sequences were acquired independently by MRI technologists instead of SSH sequences. Those rapid MRIs involving non-SSH sequences are denoted as such in the text.

Pre-operative MRI preparation was provided by child life services along with intra-procedural techniques such as play therapy or movie watching for some patients. Parental and nursing assistance for positioning were also available. No anesthesia was present to provide any form of sedation.

### Procedural time

The total procedural time of rapid MRI was calculated to include all aspects of patient positioning and transition on or off the MRI table in the imaging suite. This manner of calculation is necessarily longer than the acquisition time of MRI scanning reported elsewhere [14] since scanning cannot begin until a patient is positioned. This method accounts for the possibility that full MRI with sedation may make patient positioning more time efficient to offset known differences in scan acquisition time.

### Statistics

All statistical calculations were performed in RStudio 2022.071 Build 554. Statistical comparisons in Table 1 were performed with the Mann–Whitney *U* test for age, after determining non-normal distribution with a Shapiro test. Chi-squared tests were used to compare differences in GMFCS 1–3 vs 4–5 and gender distribution. Sensitivity reported in Table 2 was calculated as the total number of patients whose MRI was able to reveal the level of conus level by radiology read, divided by the total number of patients in the respective cohort for whom MRI was performed.

**Table 1:** Patient characteristics by MRI cohort

*Patient* a*ttribute*	*Rapid MRI cohort*	*Full MRI cohort*
*Mean age (25th, 75th quartile)*	9 (6, 11)	10 (7, 11)
*GMFCS 1–3*	37 (46.8%)	13 (61.9%)
*GMFCS 4–5*	42 (53.2%)	8 (38.1%)
*Male*	52 (65.8%)	13 (61.9%)
*Female*	27 (34.2%)	8 (38.1%)
*Total*	79	21

The mean age, Gross Motor Function Classification System (GMFCS) score, sex, and total number of patients in rapid and full MRI cohorts are presented for comparison. There was no significant difference between age (*p* = 0.55), GMFCS distribution (*p* = 0.32), or gender (*p* = 0.93) between cohorts

**Table 2:** Conus level sensitivity

***MRI cohort***	***Conus identified***	***Sensitivity***
*Full MRI*	20/21	95.20%
All rapid MRI	77/79	97.40%
SSH rapid MRI	57/59	96.60%

The sensitivity for conus level localization is presented as a fraction of the total of each cohort and a percentage. The *full MRI* cohort had sedation for all scans. The *all rapid MRI* cohort includes 59 patients with the single-shot (SSH) MRI acquisition (see “Methods” section) as well as 20 patients with non-single-shot (non-SSH) acquisition) for a total of 79 patients in the cohort. The *SSH rapid MRI* cohort is subdivided out from the all rapid MRI cohort for further comparison as these scans use the SSH rapid acquisition technique described in detail in “Methods” section. Full MRI, all rapid MRI, and SSH rapid MRI sensitivities were compared using a Fisher’s exact test which found no significant differences (*P* ~ 1.0)

For the statistical purposes of this paper, the adequacy of the rapid MRI was determined by the radiologist’s ability to report the level of conus. Radiology report was chosen to remove any surgeon/author bias to influence the adequacy of the rapid MRI sequence in this report. There were no instances when the intra-operative impression was different from the planned procedural level.

Differences in sensitivity were calculated between all rapid MRI, SSH rapid MRI and full MRI using Fisher’s exact test.

Total procedural times were compared using the non-parametric Wilcoxon Rank Sum test.

## Results

Patients who underwent single-level laminoplasty for SDR were reviewed, of whom *N* = 100 had documentation of an MRI for pre-operative imaging. Of those reviewed, 79 had a rapid MRI, and 21 had a full MRI with sedation. Of the 21 patients who received a full MRI with sedation, 20 were due to interdisciplinary spasticity clinic recommending full MRI brain imaging, as well as spine, to rule out structural etiology of spasticity. One patient got a full MRI after motion artifact affected the rapid MRI quality. There was a mix of patients whose indication for SDR was functional improvement of ambulation and those whose indication was for therapeutic reduction in spasticity. The age, sex, and pre-operative Gross Motor Function Classification System (GMFCS) score distribution is summarized in Table 1. There were no significant differences for these attributes between cohorts.

**Fig. 1:**
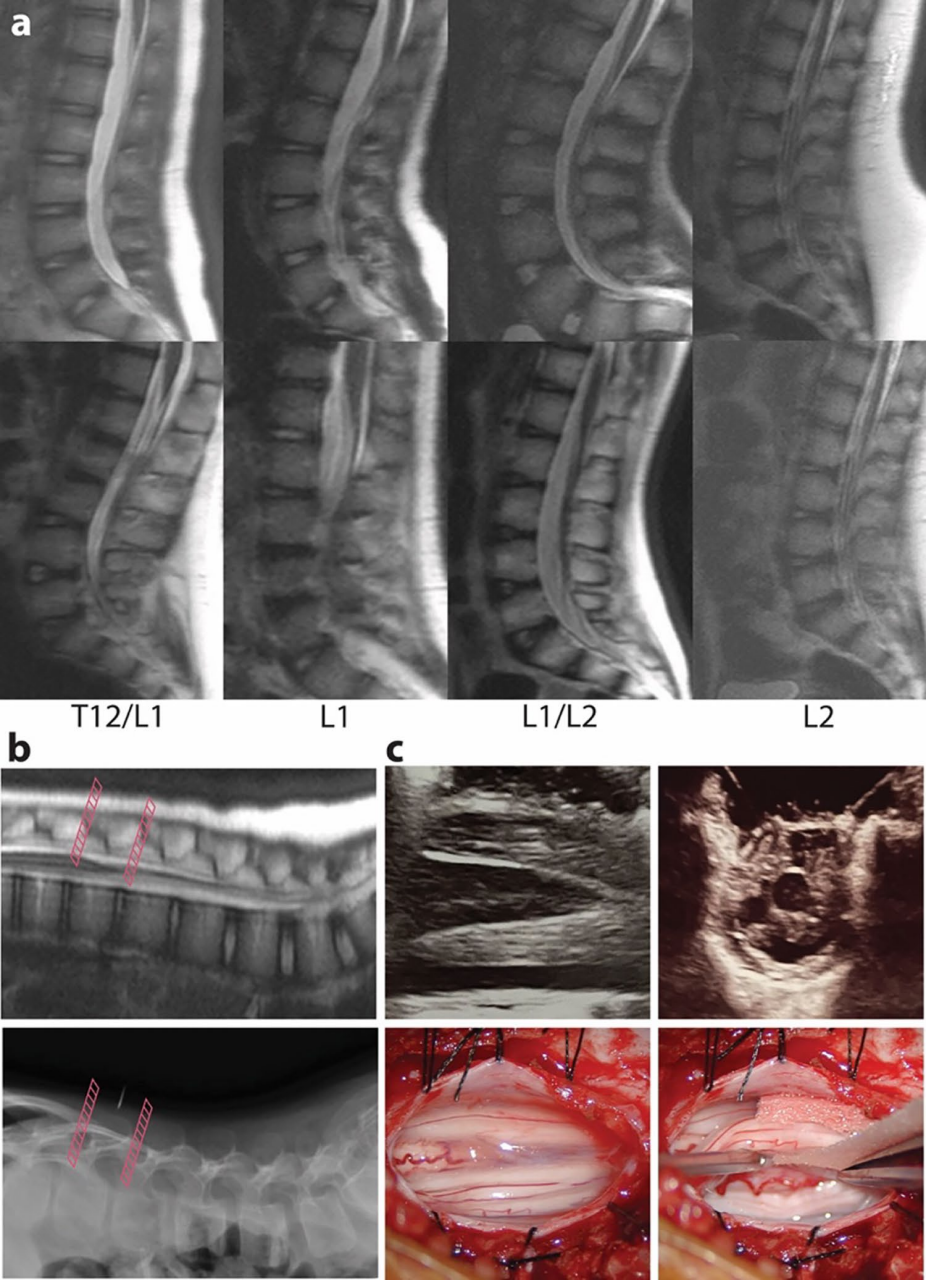
Rapid MRI of the lumbar spine.** a** Representative examples of rapid MRI of the lumbar spine for conus localization. Sagittal SSH T2 images were chosen from 8 individual patients with conus localized to levels T12/L1–L2. Each top and bottom pair of separate patient images have conus localized to the level listed below them (i.e., each column represents 2 separate patients with conus at the same level. **b** Sagittal SSH T2 image illustrating clear conus localization over the L1 vertebral body (top). Pink cross-hatched lines approximate rostral and caudal limits of exposure from a one level laminoplasty. The cross-hatching represents approximate areas that will be better visualized if the superior spinous ligament is divided at the respective mark (i.e., rostral or caudal end of the laminoplasty level). Intra-operative lateral x-ray (bottom) is used to confirm correct level when compared with pre-operative rapid MRI. **c** In a separate patient from the imaging in (**b**), sagittal (top left) and axial (top right) intra-operative ultrasound images of conus localization during single-level laminoplasty, prior to dural opening. Intra-operative photographs of the conus (bottom left) of the same patient for which the ultrasound is presented, showing sufficient working room for selective dorsal rhizotomy

Representative sagittal T2-weighted images of rapid MRIs for conus localization demonstrate the range of image quality for these rapid sequences (Fig. 1a) with a range of conus levels. Comparison of one such scan with the associated intra-operative localizing x-ray (taken prior to laminoplasty) demonstrates the stepwise localization of the conus from pre-operative rapid MRI to intra-operative imaging (Fig. 1b). Following laminoplasty for dural exposure, the conus level can be confirmed by intra-operative ultrasound (Fig. 1c). A further discussion of surgical technique and workflow is found in the “Discussion” section.

There was no significant difference in sensitivity for conus level localization (Table 2) comparing rapid MRI imaging vs full MRI. Of the 79 patients with rapid MRIs, 20 were cooperative enough to include high-resolution, non-SSH sequences (i.e., 59 SSH and 20 non-SSH). Regarding the two patients whose rapid MRI lumbar spine did not localize conus, both were in scans of only SSH sequences. One was due to motion artifacts, and the other was due artifacts arising from spinal hardware. The single patient whose full MRI did not localize conus was due to artifact from spinal hardware.

**Fig. 2:**
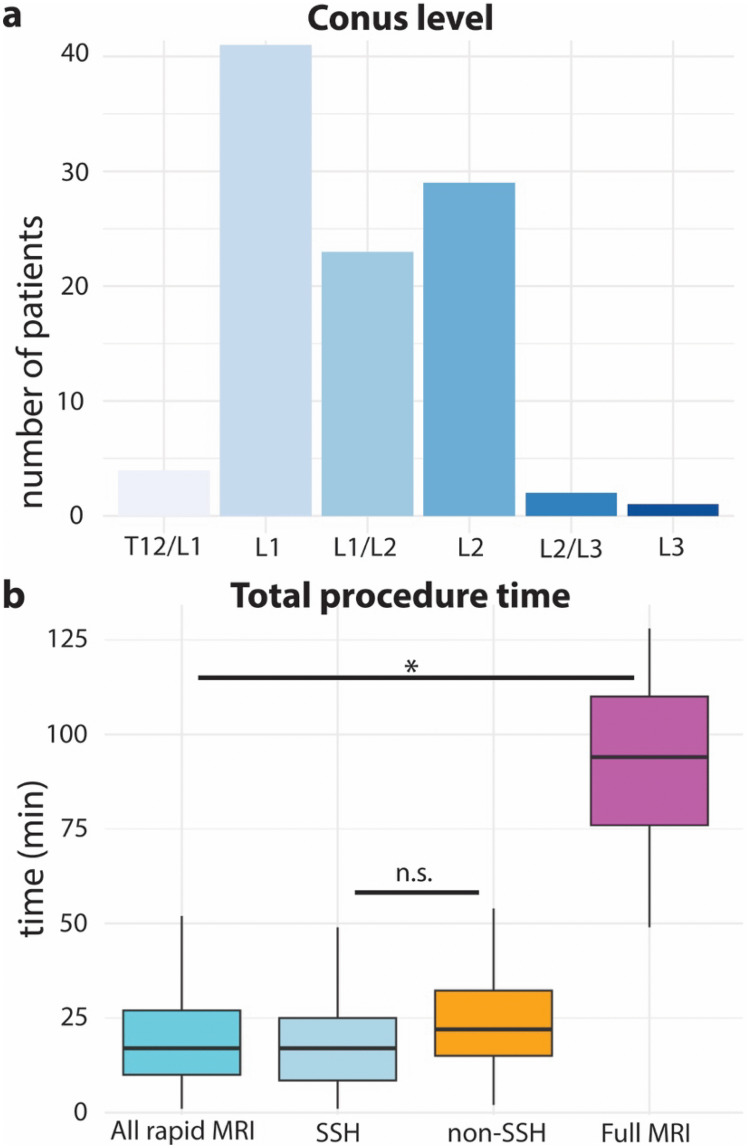
Conus level and procedure time.** a** Conus level localization for the entire cohort. **b** Box and whisker plot of total procedure time compared between all rapid MRI, single-shot (SSH) rapid MRI, non-single-shot rapid MRI (non-SSH), and full MRI with sedation (full MRI). Boxes represent the 25th to 75th quartiles and black lines within boxes represent median values. The *all rapid MRI* cohort is subdivided into those with only single-shot (SSH) sequences and those with higher resolution non-SSH sequences added during acquisition in cooperative patients (non-SSH). Rapid spinal MRI studies were significantly shorter than full MRI studies (*Wilcoxon Rank Sum, *P* = 8.0 × 10^−12^). There was no statistical difference between the procedural time of SSH and non-SSH rapid MRI studies (Wilcoxon Rank Sum, *P* = 0.10). n.s., no statistically significant difference; *statistically significant difference

The distribution of conus levels ranged from T12/L1 to as low as L3 (Fig. 2a). Of note, patients with an abnormally low conus were evaluated for evidence of tethering, but a full discussion of tethering evaluation is beyond the scope of the present work.

The total procedural time of rapid MRI was calculated to include all aspects of patient positioning and transition on or off the MRI table in the imaging suite. As mentioned in the “Methods” and “Discussion” sections, this procedural time will be longer than scan acquisition time reported elsewhere [14] for similar rapid MRI sequences. Mean total procedure time for rapid MRI was 21.5 (median 17) min. Mean time for full MRI with sedation was 91.2 (median 94) min (Fig. 2b).

It is possible that the modified rapid MRI protocols which included one or two high-resolution non-SSH sequences for cooperative patients (while still avoiding sedation) involve significantly different procedure time. Separating out the 59 SSH and 20 non-SSH procedure times (Fig. 2b) did not show a significant difference in procedure time.

## Discussion

Selective dorsal rhizotomy is supported by level I evidence to improve spasticity, function, and quality of life in select patients with cerebral palsy [1, 2]. While techniques for performing SDR vary in terms of levels exposed, those involving a 1- or 2-level exposure often use a pre-operative MRI to delineate the conus level [4, 6, 16].

Rapid sequence MRI is an established tool for cranial imaging, while avoiding sedation in a pediatric population. Its use in pediatric spine is less well-described. Here, we demonstrate a use-case scenario for routine rapid MRI of the lumbar spine for pre-operative conus localization in patients selected to undergo single-level laminoplasty SDR. Rapid MRI of the lumbar spine showed comparable accuracy for conus localization compared to a full MRI with sedation.

### Surgical technique and workflow

Obtaining accurate conus level pre-operatively is required for a single-level laminoplasty. Prior to positioning, the relevant imaging showing the conus level is displayed in the operating room. Once the patient is positioned prone and neuromonitoring electrodes are in place, a localizing x-ray is obtained to plan the incision at the level optimal for exposing the conus. The level is again confirmed intra-operatively before laminoplasty (Fig. 1b, lower panel).

The laminoplasty technique used involves a single division of the interspinous ligament either rostral or caudal to the superior spinous process of the laminoplasty level. Once divided, the lamina is reflected laterally or in the direction of the intact interspinous ligament. As such, whichever side (rostral or caudal) of the laminoplasty that is divided will have a slightly lengthened working area relative to the intact ligament’s side. This extra amount of working room is represented visually by the pink cross-hatched lines in Fig. 1b.

Following laminoplasty and exposure of the dura, ultrasound can be used to confirm localization of the conus prior to durotomy (Fig. 1c, top panels). The dura is opened and tacked up widely (Fig. 1c, bottom panels) such that dorsal and ventral bundles can be distinguished relative to the conus/cord. Dorsal–ventral distinction is then confirmed with direct electrical stimulation for electromyographic (EMG) thresholds [16]. When closing, the interspinous ligament is re-approximated at the single (rostral or caudal) side of the laminoplasty that was sectioned, since the opposite side of the ligament is left intact. This is followed by re-approximation of the laminoplasty with absorbable fixation plates.

### MRI procedure

The difference between rapid MRI sequences and conventional (i.e., non-rapid or non “single-shot”) MRI sequences is that for rapid MRIs, the images are each acquired individually in rapid succession, with each individual image typically acquired in one second or less [15, 17]. Since each image is separately acquired, if there is movement during the ~ 1 s of data acquisition for a particular image, this will only affect that specific image and leave other images acquired before and after the movement unaffected. Conventional MRI sequences are acquired over the course of minutes, such that any motion during the minutes-long acquisition will result in motion artifacts. Therefore, rapid MRI can be obtained for conus localization in this patient population without sedation.

Non-anesthetic techniques such as play therapy, parent participation, and other forms of child life preparation may make the MRI-scanner less axiety-producing [18, 19]. While our patients tended to be young with cerebral palsy, rapid MRI may not be possible in older patients with severe behavioral difficulties that cannot stay positioned in the scanner even with parent, caretaker, or MRI nursing assistance.

The present study reports total procedural time recorded by the MRI technologist. This typically involves the time taken for all aspects of the scan, including patient positioning. As such, these MRI procedural times will necessarily be longer than the times reported elsewhere for sequence acquisition [14, 15]. One advantage of reporting total procedural time is that it may give a more accurate representation of the time that an MRI scanner will be occupied (21 vs 91 min). This may in turn be useful from a logistical workflow perspective. In addition, procedural time will likely be congruent with the time experienced by patients, parents, and caregivers of pediatric patients.

Single-shot rapid MRI techniques, also used in fetal MR examinations [17], allow for sub-second acquisition per image to minimize motion artifacts by effectively freezing low amplitude motion. The tradeoff is decrease in spatial resolution. While a prior study has shown that rapid MRI of the spine can visualize relevant pediatric intradural pathology such as syrinx [15], these techniques are not meant as a replacement for full conventional MRI when the goal is to rule out all other pathology. Since accurate localization of the level of the conus does not require the highest resolution imaging, single-shot techniques are well-suited and allows for successful imaging without sedation.

Using a rapid spinal MRI to resolve conus level has the advantage of avoiding additional anesthesia, intubation, and excess time that comes with obtaining a full MRI spine. The shorter duration of the procedure can also impact the overall patient and family experience.

## Conclusion

A single-level laminoplasty approach to selective dorsal rhizotomy may be performed with a rapid spinal MRI for conus localization to avoid sedation or intubation procedures required for conventional MRI in many pediatric patients with cerebral palsy.

## Data Availability

No datasets were generated or analyzed during the current study.
